# Experiences of a new cadre of midwives in Bangladesh: findings from a mixed method study

**DOI:** 10.1186/s12960-020-00505-8

**Published:** 2020-10-06

**Authors:** Rashid U. Zaman, Adiba Khaled, Muhammod Abdus Sabur, Shahidul Islam, Shehlina Ahmed, Joe Varghese, Della Sherratt, Sophie Witter

**Affiliations:** 1 Health and Nutrition Portfolio, Oxford Policy Management, Level 3, Clarendon House, 52 Cornmarket Street, Oxford, OX1 3HJ United Kingdom; 2 Bangladesh Office, Oxford Policy Management, Dhaka, Bangladesh; 3 Dhaka, Bangladesh; 4 Mitra and Associates, Dhaka, Bangladesh; 5 Department for International Development, Government of United Kingdom, Dhaka, Bangladesh; 6 Faculty of Public Health and Tropical Medicine, University of Jazan, Jazan, Saudi Arabia; 7 Wotton under Edge, United Kingdom; 8 Institute for Global Health and Development, Queen Margaret University, Edinburgh, United Kingdom

**Keywords:** Bangladesh, Midwives, Health workforce, Health systems

## Abstract

**Background:**

Bangladesh did not have dedicated professional midwives in public sector health facilities until recently, when the country started a nation-wide programme to educate and deploy diploma midwives. The objective of the findings presented in this paper, which is part of a larger study, was to better understand the experience of the midwives of their education programme and first posting as a qualified midwife and to assess their midwifery knowledge and skills.

**Methods:**

We applied a mixed method approach, which included interviewing 329 midwives and conducting 6 focus group discussions with 43 midwives and midwifery students. Sampling weights were used to generate representative statistics for the entire cohort of the midwives deployed in the public sector health facilities.

**Results:**

Most of the midwives were satisfied with different dimensions of their education programme, with the exception of the level of exposure they had to the rural communities during their programme. Out of 329 midwives, 50% received tuition fee waivers, while 46% received funding for educational materials and 40% received free accommodation. The satisfaction with the various aspects of the current posting was high and nearly all midwives reported that a desire to work in the public sector in the long run. However, a significant proportion of the midwives expressed concerns with equipment, accommodation, transport and prospect of transfers. The scores on the knowledge test and self-reported skill levels were varied but reasonably high.

**Conclusion:**

While the midwives are highly motivated, satisfied with many aspects of their current jobs and have adequate knowledge and skills, there are some bottlenecks and concerns that, if unaddressed, may derail the success of this programme. To capture the career progress of these midwives, additional research, including a follow-up study with the same cohort of midwives, would be beneficial to this programme.

## Background

There is significant global evidence that women and their newborns receiving midwife-led care are less likely to develop complications and that scaling up professional midwifery can avert maternal deaths, stillbirths and neonatal deaths [1, 2]. Research also shows that midwifery is associated with improved health outcomes and efficient use of resources when it is provided by midwives who are well-educated, trained, licensed, regulated and integrated into a health system with effective teamwork, referral mechanisms and sufficient resources [3]. However, in 2014, only 4 out of 73 high-burden countries (countries with 92% of global maternal and neonatal mortality) had a midwifery workforce that was adequate to meet the needs and there is a consensus that more midwives are needed to improve maternal and newborn survival [4, 5]. Bangladesh is one of those countries which lacked dedicated midwives in its health workforce [6–8].

Various initiatives have been taken in Bangladesh over the last four decades to increase the number of healthcare workers with the competencies to assist women and newborn to make pregnancy and childbirth safer. Historically, the nursing colleges have attempted to integrate and provide supplementary midwifery education to nurses to produce nurse-midwives to work in health facilities [9]. Community maternal and child health services are provided mainly by family welfare visitors (FWVs), who are trained in the country’s public health administration and training institutions, named family welfare visitors’ training institutes (FWVTIs), under direction of the Family Planning Directorate (FPD).

 In the 1980s, the Ministry of Health and Family Welfare (MoHFW) provided a short training to traditional birth attendants (TBAs) to produce more than 50,000 trained traditional birth attendants (TTBAs) to assist women in their communities and supplement the work of the FWVs, especially in hard to reach areas. The programme did not achieve its intended results as studies have shown that the TTBAs continued to adhere to their traditional practices [10, 11]. Similar initiatives of training TBAs have failed in other countries as well and the World Health Organization has cautioned against plans to simplify and delegate tasks [12, 13]. In 2003 responding to the global call to provide all pregnant women with a skilled birth attendant and recognising that vast numbers of women still gave birth outside of a health facility, the MoHFW embarked on a programme to provided 6 months training to community health workers and produced around 13,000 community skilled birth attendants (CSBA). Despite large investment in this programme, the impact could not be established and there were confusions with their dual role of providing community health services and as skilled birth attendants (SBA), in addition to the questions of competency in fulfilling the definition of a SBA and the low uptake of services by the community [14, 15]

In 2010, at the 65th General Assembly of the United Nations the Prime Minister of Bangladesh announced producing and deploying dedicated midwives as an attempt to have a permanent solution to the shortage of SBAs [4, 6]. Subsequently, the curriculum and the regulatory frameworks for the diploma midwifery education and mentorship programme were developed with support from the development partners [16, 17]. The curriculum has been designed to incorporate general courses (Science and English), foundation courses (such as anatomy and pharmacology) and professional courses (from newborn complexities to maternity experience) [18]. In September 2018, 55 education and training institutes (38 government and 17 private) had 1565 available placements (975 government and 590 private) to produce diploma midwives, while the government created 2996 midwifery service posts in 1733 primary healthcare facilities across the country. In 2018, MoHFW recruited 1143 licensed midwives and deployed them in 342 health facilities (Personal communications, MoHFW and United Nations Population Fund (UNFPA)).

The objective of this paper, which is part of a larger study, was to better understand the experience of the midwives in their education programme and their first posting and to assess their knowledge and skill.

## Methods

### Overall design

There were two components in this mixed method research: focus group discussions (FGDs) and a survey of midwives. The tools were developed based on the International Confederation of Midwives (ICM) Global Essential Competencies for Midwifery Practice statements [19]. The survey included a knowledge and skills test. The knowledge test tools were adapted from previous studies and were based on clinical scenarios of antenatal care (ANC), delivery care, postnatal care (PNC), newborn care and family planning services [20]. The skills were self-assessed for various clinical procedures.

### Sampling

A total of 1143 midwives, including 985 obtaining diploma from public and 158 from private institutes, were deployed in government health facilities in August 2018. Based on this, the study required a participation of 288 midwives in order to obtain results accurate to within ± 5% at the 95% confidence level and a prevalence of 50%. Simple random sampling was used without any clustering, due to the distribution of the midwives, and therefore, the design effect was 1. Anticipating a response rate of 85% and likely levels of attrition in future follow-up surveys, we over-sampled and reached out to 331 midwives for the quantitative research component. The sample was representative for the entire cohort of the midwives who have joined the public service in the first two batches. The sample size was large enough to detect some statistically significant differences between the key groups of research interest. These include the type of the education institutes (public versus private) and the location of job (urban versus rural). Since a relatively small proportion of the midwives were from the private education institutes in the overall sampling frame, we over-sampled the midwives from the private institutes in order to have sufficient sample in that strata that would allow us to allow comparison between the midwives educated in public and private institutes.

For the qualitative element, six FGDs were conducted with 5–10 midwives or midwifery students per FGD and 43 midwives participants in total; three in the capital Dhaka city and the remaining in Sylhet, Manikgonj and Matuail. FGDs included professional midwives recently placed at public sector health facilities or private/NGO run health facilities and those currently in the diploma course to be midwives in public or private institutes.

All respondents took part voluntarily and informed oral consent was taken from each individual before the interviews and FGDs took place. Confidential information has been excluded from the datasets to maintain anonymity.

### Data collection

All the tools were designed and pretested by the researchers. The tools that we used for the interviews were translated in Bangla, which is the native language of all the enumerators and respondents. Ethical clearance was taken from the ethical research committee based at the Institute of Health Economics (IHE) at University of Dhaka and from the Ethical Review Committee of OPM. Experienced quantitative and qualitative enumerators were then recruited and undertook in-depth training and piloting of the tools. Data were collected between September and December 2018. Quantitative data collection took place first, using structured questionnaires filled using computer-assisted personal interviewing (CAPI). Semi-structured topic guides were used for the FGDs, which were conducted by an interviewer and rapporteur and were recorded and then later transcribed.

### Data analysis

The quantitative data were analysed using SPSS and Stata. Since the midwives from the private institutes were over-sampled, sampling weights were used during analysis to reflect the proportional representation of the sampling population. All the quantitative data presented in the “Results” sections are based on the weighted sample. We used descriptive statistics and tested statistical significance using a two-sample test of proportions at 95% confidence, where *p* value of < 0.05 was considered statistically significant. To understand the costs associated with the education programme, Bangladeshi Taka (BDT) was used as the currency in the questionnaire and an exchange rate of 1 Great Britain Pound (GBP) equals 110 BDT was used as the exchange rate during the analysis. We undertook thematic analysis using the qualitative data using the framework method [21].

## Results

### Study population

Out of 331 sampled midwives, we interviewed 329, which is a response rate of 99.4%. The high response rate is mainly because prior contact was made with the midwives over the phone to explain the study and set a face to face interview date.

The mean age of the sampled midwives was 24.5 years with the standard deviation (SD) of 1.76. Among all, 58.2% were posted in urban and 41.6% in rural health facilities. The mean (SD) distance from work for those living outside the premises was 6.8 (10.9) km. Table 1 outlines the description of the study population.

**Table 1:** Description of the study population

Characteristics	Proportion (%)
Locality of the health facility	
Urban	58.4
Rural	41.6
Accommodation	
Accommodation provided by the facility	55.3
Own accommodation	39.5
Rented accommodation	5.2
Mode of travel to work	
On foot	86.9
Public transport	13.1
Transport provided by health facility	2.7
**Total number of respondents (*N*)**	**329**

### Motivation to enter the midwifery education programme and the experiences

Out of 329 midwives interviewed, just over half (55.7%) were encouraged by their parents to become a midwife, while others were encouraged by other family members (18.7%) or relatives (14.1%). Helping people was the most commonly (82.5%) stated motivating factor, followed by suggestions from family members and relatives (6.4%), better social status (2.1%) and to have a job with greater responsibility (2.1%). Results from the qualitative research suggests that many of the midwives were initially drawn to pursuing a nursing degree, but were then encouraged by relatives and family members to join this relatively new programme that specialised in maternal and newborn care. In some cases, leaflets promoting the diploma were circulated in the community, which caught the attention of many. However, several participants admitted not knowing much about the midwife profession when submitting applications or even during the interview for the admission.

Half of the midwives received waiver of the tuition fee. A significant proportion of the midwives also received funding for education materials (45.9%), accommodation (40.1%) and other expenses (26.0%). Among all (82.0% midwives paid a one off admission fee, with an average of BDT 5500 (GBP 50.00). Almost all the midwives spent some amount for living costs, the average being BDT 3000 (GBP 27.27) per month.

**Fig. 1:**
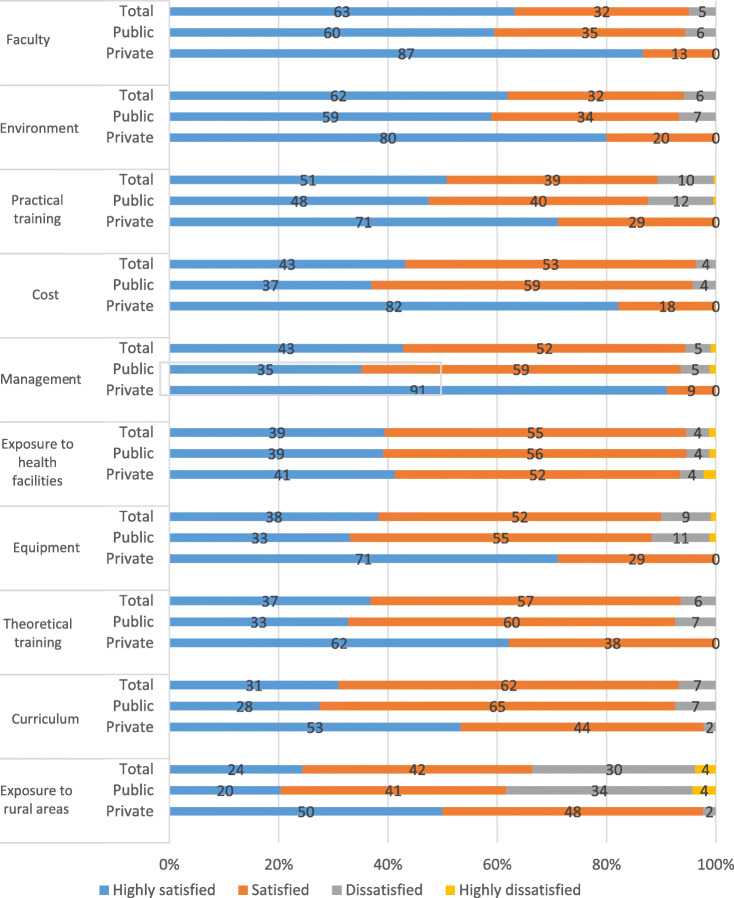
Satisfaction level with the education programme

Satisfaction with the various components of their programme was self-assessed by the respondent on a scale of 1 to 4, with 1 being highly satisfied to 4 highly dissatisfied, which was later merged into simpler groups: satisfied or dissatisfied. The dimensions of the assessment included the following: management of the course, instructors, curriculum, available equipment, theoretical and skills development and practical exposure to rural areas and health facilities. Data in Fig. 1 presents overall high satisfaction among the midwives, with slightly higher levels for midwives educated at private institutes when compared to those at public institutes. The main difference between the two groups is that 38.4% of those educated in the public institutions reported they were unhappy with the level of exposure they had to rural communities during their programme. The reported differences in satisfaction between public and private institutes were found to be significant for exposure to health facilities (*p* < 0.01), cost (*p* < 0.05) and curriculum (*p* < 0.05).

Probing during FGDs revealed some other areas of dissatisfaction. As midwifery was a new course in public institutions, midwifery students reported facing discrimination from nursing students, faculties and authorities. One of the responded mentioned:

“Since our institution was originally for nurses, our tutor gave us very little time and remained busy with the nursing students” 

- Midwifery student from a public institution

Public institutions attached to teaching hospitals had problems in ensuring the required clinical component, as the midwifery students needed to compete with medical students, intern doctors, doctors in post graduate training and student nurses for hands-on practice. In the medical hierarchy, midwifery students, being new, were at the bottom and thus faced challenges in gaining appropriate support and practice opportunities.

Private institution students had better theoretical education, community exposure, teaching and learning aids and accommodation. However, since many of these private institutes did not have their own hospitals, the students were placed in public facilities for clinical and skills development and as such experienced the same problems as mentioned above. In addition, at times there would also be competition between student midwives from public and private institutes as both were placed in for the same clinical areas, with students from the public institutions getting preference. However, the private institutes were better equipped with dummies and kits and skills development rooms, whereas in the public institutions, facilities for the clinical and skills development and many other teaching and learning tools were unavailable. The students who studied in Bangla in the primary and secondary schools also initially struggled to adapt to English as the medium of study, but eventually managed to cope with the mode of communication, with extra English language teaching.

### Current post and tasks

The current employment was the first job for most (72.6%) of the midwives and the current facility was the first posting for nearly all (96.7%) respondents. A majority (60%) of midwives were born in the districts in which they were posted. More than half of them (55.2%) did not have any preference about their posting, whereas a third (36%) reported that their current posting was not their preferred workplace.

**Fig. 2:**
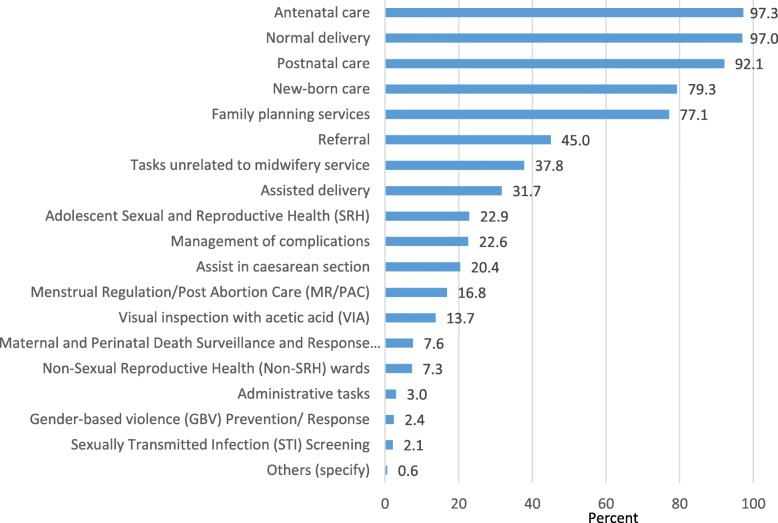
Tasks performed by the midwives at the health facilities

Less than 3 months into the posts, the midwives reported performing some but not all of the key tasks that they are supposed to carry out at their current workplace, including ANC, normal delivery, PNC, newborn care and family planning services (Fig. 2). Less than half had yet worked on core areas such as referral, assisted delivery, adolescent sexual and reproductive health and managing delivery complications. More than a third (38%) of the midwives reported performing tasks unrelated to midwifery services (e.g. nursing). In the FGD, some midwives reported filling in for nurse duties in their absence, attending to patients in the general ward and giving child vaccines and expressed discomfort in dealing with male patients. As a result, some complained of being unable to concentrate on the specific tasks for which they were qualified for. Most of the midwives reported their workload as manageable, with 78.4% reporting that “it is just right”, 17.0% as “sometimes it is difficult to manage”, 4.3% as “I can do more” and 1 midwife considered it as “completely unmanageable”. There was no significant difference in reported workload between urban and rural facilities, with 83.1% of the midwives posted in urban facilities reported to having the capacity to do more or workload being just right as compared to 82.0% of their rural counterparts (*p* = 0.787).

**Fig. 3:**
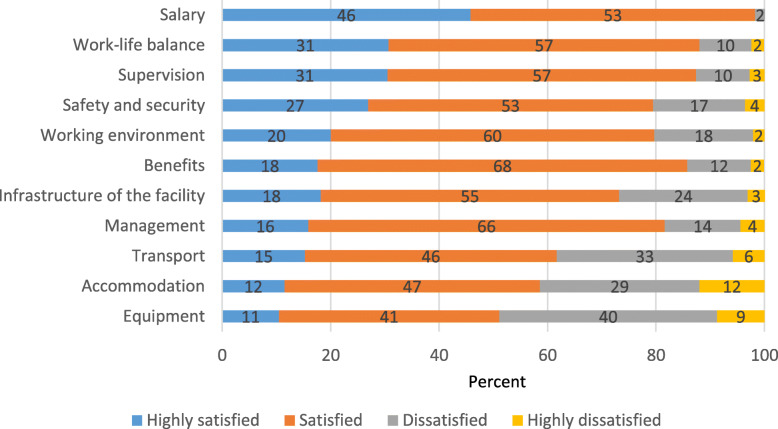
Satisfaction with the current job

Satisfaction levels of the current job were self-assessed by the midwives using the same scale as above. As evident in Fig. 3, nearly all midwives were satisfied with their income, and the majority were satisfied with the work-life balance, supervision, benefits, management, working environment and security and safety. Dissatisfaction was mostly around lack of sufficient equipment needed to conduct the tasks of a midwife, but also regarding accommodation, transport and infrastructure of the facility. This was also evident from the FGDs, where it was reported that limited medical kits were a challenge during community counselling, as was lack of appropriate accommodation for counselling as public health complexes were overcrowded.

There are no significant differences (*p* > 0.05) in satisfaction between the midwives posted in urban and rural areas in most of the areas except accommodation and work-life balance. On accommodation, the majority (68%) of the midwives posted in rural areas are satisfied compared to just half (51%) in urban facilities (*p* = 0.002). In contrast, 18% midwives in rural facilities reported not having a proper work-life balance, compared to 7% in urban facilities (*p* = 0.003).

### Preferences, motivations and concerns

Nearly all midwives (99.7%) reported that the public sector is their long-term preferred employer. From the discussion with professional midwives that took place in a private health complex, many of the participants were waiting and hoping to be given an opportunity to join the public workforce. Nearly two thirds of the survey respondents preferred working in urban health facilities. Sub-district health complexes (37.9%) and district hospitals (31.5%) were the two top preferred workplaces, with less preference (5.5%) for work at union-level facilities. Almost all midwives wished to pursue higher education (nearly half mentioning wishing to study for a PhD while others mentioned a bachelor’s or a master’s degree) in future, and three quarters of them would like to migrate abroad at a later stage of their career (Table 2).

**Table 2:** Long-term preference of the midwives

	%
**Locality**	
Urban	66.5
Rural	31.1
No preference	2.4
**Sector**	
Public/ government sector	99.7
Private sector	0.0
NGO sector	0.3
Others	0.0
No preference	0.0
**Facility type**	
Tertiary hospitals	7.0
District hospitals	31.5
Maternal and child welfare centres	17.7
Upazila health complexes^a^	37.9
Union sub-centres^b^	5.5
Family welfare centres^b^	0.3
Others (specify)	0.0
No preference	0.0
**Wish to migrate abroad**	
Yes	74.8
No	25.2
**Desire to undertake higher education**	
Yes	99.7
No	0.3
**Number of respondents (*N*)**	**329**

^a^Upazila health complexes are primary level health care facilities at a sub-district level with inpatient and outpatient facilities

^b^Union sub-centres and family welfare centres are union (lowest administrative area) level facilities with outpatient services only run by health and family planning departments respectively

**Fig. 4:**
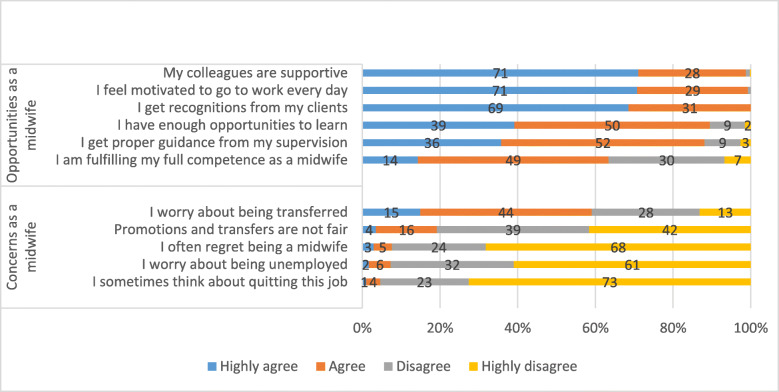
Agreement with career-related statements

The vast majority of the midwives reported being highly motivated, having enough opportunities to learn, getting proper guidance and support from the supervisors and colleagues and being recognised by the clients. However, more than one third reported that they were not yet reaching their full potential in fulfilling the role of a midwife. Most of the midwives were not worried about fairness in relation to transfers and promotions or being unemployed. Most midwives neither had regret in choosing this profession nor any plans to quit. However, 59.1% midwives reported they were concerned they would get transferred (Fig. 4). The reasons for their concerns about being transferred were not probed in the study.

### Knowledge and skills

**Fig. 5:**
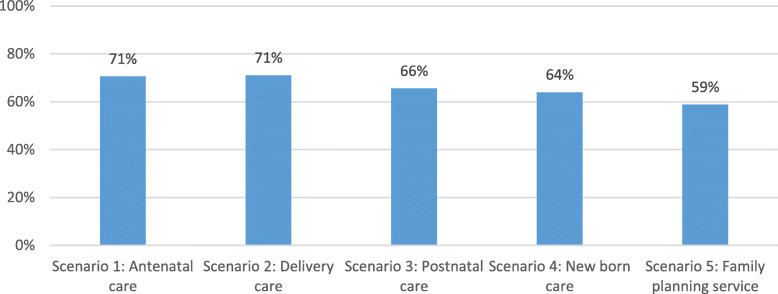
Knowledge test scores, by areas

A knowledge test was conducted in the survey, where a series of clinical scenarios that covered a wide spectrum of midwifery responsibilities were presented and then alternatives for step by step actions that were to follow provided, in order to assess the midwives’ decision-making skills and whether they knew the correct action to take. The mean score on knowledge across five areas (ANC, labour and birth care, postnatal care, newborn care, family planning) was 66.2%, with generally higher knowledge on ANC and labour and childbirth care (Fig. 5). With regard to the various steps in a consultation, the midwives scored 56% on history taking, 72% on examination, 60% on diagnosis and 75% on management.

This was followed by a self-assessment of a set of key skills covering the five main areas mentioned above, to determine whether they had learnt a particular skill either during their programme or following graduation at work, whether they had the confidence in performing the skill and then when needed and whether they had actually conducted this in the last 1 month. If they reported they had not practiced the specific skill, there was further probing to understand why they had not practiced it yet. A diverse set of 30 skills over the five areas were identified, such as the ability to correctly calculate an expected date of delivery (EDD), measure the fundal height, recognise and manage low haemoglobin in pregnant women, recognise the onset of labour, recognise and manage birth asphyxia, and provide client-focused family planning advice and services to managing postpartum haemorrhage and eclamptic fits (see Table 3).

**Table 3:** Self-assessment of midwifery clinical skills

	Learnt this skill in the education programme (%)	Learnt this skill at work (%)	Confident to perform this skill at work (%)	Performed this skill in the last month %
Taking an antenatal history	99.6	99.6	99.1	97.2
Calculating expected date of delivery (EDD)	100.0	99.5	100.0	98.3
Measurement of uterine size with tape	99.1	96.3	99.1	88.2
Provide individual counselling for preparing for birth/ making birth plan and emergency plan	99.9	99.5	99.6	95.9
Recognition of low haemoglobin in pregnant woman	98.2	95.5	95.8	82.0
Management of anaemia in pregnancy	99.6	97.7	99.5	84.4
Provide group counselling to the community on healthy pregnancies and safe motherhood	94.3	83.9	97.4	60.3
Identify signs of the onset of labour	100.0	99.6	99.6	95.5
Determination of foetal position by abdominal examination	98.4	97.2	98.5	88.6
Listening to the foetal heart sounds	100.0	100.0	99.9	97.2
Use of partograph	99.1	89.4	98.1	69.9
Identify the signs of second stage of labour	99.6	100.0	100.0	95.1
Manage second stage of labour	100.0	99.6	99.6	93.3
Natural (with no oxytocin) management of 3rd stage of labour	98.2	94.8	96.3	78.6
Inspection of placenta and membranes	100.0	99.6	100.0	92.0
Perform manual removal of placenta	99.4	95.1	97.2	63.1
Suture perineum	97.9	98.4	99.0	80.4
Assess APGAR scores	100.0	99.5	100.0	94.9
Helping baby take first breath	99.9	99.4	100.0	85.4
Assist in immediate breastfeeding	100.0	99.6	100.0	95.3
Perform newborn eye care	98.6	96.4	98.2	84.2
Recognise uterus is well contracted immediately postpartum (after birth)	99.9	98.6	99.6	94.4
Provide client-focused postnatal Family Planning counselling	98.5	98.9	98.5	93.5
Examine newborn	99.0	99.1	99.9	92.9
Diagnose postpartum haemorrhage	100.0	96.5	99.1	57.9
Manage postpartum haemorrhage	99.6	97.9	99.6	50.8
Diagnose infection in the newborn and give appropriate immediate care for newborn	99.1	89.2	95.4	50.6
Diagnose sepsis in postpartum women and give immediate care	99.6	91.2	96.7	43.2
Recognise women with eclamptic fits	100.0	87.1	98.1	26.0
Manage eclamptic fits including giving magnesium sulphate	99.5	82.0	97.0	24.2
**Number of respondents (*N*)**	**329**	**329**	**329**	**329**

The midwives scored themselves highly in self-reported clinical skills against various clinical procedures. They reported learning all the key skills during their education programme as well as later at their workplace, with most of them reporting they felt confident to perform those at work. However, in most cases, it was a lack of opportunity that prevented the midwives from performing those tasks during the past month. In particular, shortage of cases did not permit the practice of more complex skills, such as diagnosing or managing postpartum haemorrhage. In other instances, midwives were unable to perform some tasks such as using a partograph or providing counselling, as seniors denied them permission to do so (Table 3). As mentioned repeatedly in FGDs, in the presence of doctors, the midwives are unable to have hands-on experience of assisting at a normal delivery during clinical practice. In most cases, they just observe from afar and, at times, were only permitted to assist by handing over equipment to the physician. When on night duty, midwives get more opportunities to practice, but cases were relatively far less in number than in the daytime.

## Discussion

This paper is a small component of a larger study to outline the initial status of a national effort for scaling up professional diploma midwives in Bangladesh as a new and dedicated health workforce. It is too early to comment on whether scaling up diploma midwives is likely to achieve the expected outcomes. However, there is evidence that there are women willing to enter and remain in this profession. This is important as evidence exists to show that a skilled health workforce dedicated to addressing maternal and neonatal health can have important impacts [22, 23]. Previous studies have also suggested that there is high political commitment for scaling up the midwifery programme in Bangladesh and that the programme has high potential, particularly if multiple partners and stakeholders work in close collaboration [24]. Finally, our study demonstrates that a curriculum covering the internationally agreed competencies of a midwife does exist and is being implemented to produce competent professional midwives in Bangladesh.

The findings of our study are broadly consistent with the social, professional and economic barriers highlighted in other studies [25, 26]. While the midwives are highly motivated and keen to make a difference, an enabling working environment is critical to their success and is still to be secured. It is concerning that 38% of the tasks reported by the midwives are unrelated to midwifery. A previous study in Bangladesh had reported that nurses used only 6% of their time in patient care because of various health systems and social factors [27]. This indicates that there is a precedence of inappropriate use of health workforce in Bangladesh, which might affect this newly deployed workforce as well. A study conducted with government health workers in Bangladesh reported a high level of dissatisfaction, including inadequate supplies and infrastructure, bad behaviour of patients and administrative problems [28]. A Cochrane review on the factors influencing the performance of skill birth attendants in low- and middle-income countries also identified access to training and supervision, staff numbers and workloads, salaries and living conditions and access to well-equipped and well-organised health facilities as the key driving factors to keep the SBAs motivated [29]. Therefore, it is possible that the current high motivational level of the midwives may not continue if these factors are not addressed.

The results from this study regarding the mean score of the knowledge test of the midwives is positive and was much higher than reported for nurses using a similar tool, which was 25.2% in a previously conducted survey [20]. Both nurses and midwives are currently in the same level (second class officer) in public service, and from the knowledge and skills assessment, there is no reason not to see these two cadres as equal. Not only were the findings of the knowledge and skills assessment reassuring, the midwives also reported to be satisfied with their education programme. Again this is another positive finding given that a previous study highlighted some gaps in education capacity and quality assurance of the midwifery programme in Bangladesh [30]. Our results showing satisfactory clinical knowledge and a high level of self-reported confidence on clinical skills suggests that some of those early concerns have been addressed. However, it is important to be mindful that this finding could be because they have just finished their programme and started their job, and therefore have not yet full experienced all the professional and personal challenges that may face them.

While, globally, the proportion of mothers giving birth at home has significantly reduced in recent years, 53% of the births in Bangladesh still take place at home and 29% and 4% take place at private and NGO sector health facilities respectively [31]. There is evidence that women living in poor and remote settings often chose services from the private sector over the public sector [32]. The diploma midwives are being deployed at the public sector health facilities that so far cover only 14% of the total births, leaving a vast majority of the birth places uncovered with this programme. While it is possible that institutional deliveries in the public sector facilities might increase because of the deployment of the midwives, it might take some time to make the cultural changes to move away from giving birth at home. Therefore, the ability for these diploma midwives to work as close as possible with women where women live will be paramount. Further, it is not clear in the current policy documents how the midwives in Bangladesh would contribute to increasing quality skilled birth attendance for home and private sector deliveries.

There are some important limitations of this study. Firstly, we have used a self-assessment tool for clinical skills instead of complex and expensive techniques like vignettes or direct clinical observation which can assess skills more objectively. However, this approach was considered as a suitable proxy to the study team as it has been used in evaluations of midwives’ skills in some other low-resource settings [33]. Secondly, some of the concepts used in this study (for example, faculty, environment, management and supervision) are quite complex, and it is possible that not all respondents understood these concepts in the same way. We have attempted to mitigate this risk by explaining these concepts clearly to the enumerators during the training. Finally, a Likert scale could have showed more variation in the responses instead of binary “yes” and “no” response for the self-reported skills questions, However, since the module for this question was already long and since this questionnaire has been successfully used in other settings, we did not change that [19, 33].

## Conclusions

While the results presented in this paper show the new midwives deployed at the health facilities are largely motivated, some expressed dissatisfactions regarding the education programme, crucially in relation to hands-on practice and working with women at the community level; also the work many are currently performing include tasks outside and unrelated to midwifery and for which they are not appropriately educated for. The finding also indicate that the midwives seem to have a satisfactory level of knowledge and have acquired the required skills in most of the areas except history taking, but many reported not being authorised by supervisors to perform several midwifery tasks. Lack of necessary equipment and supplies at the facility is another reason for constraining the midwives from sufficiently carrying out their duties. The ambition by most midwives appears to be able to work in the public sector, though they have concerns about being transferred, this bodes well for the public health services. Many midwives however also have plans to migrate abroad in the future, raising the concern of retention.

## Data Availability

The datasets used and/or analysed during the current study are available from the corresponding author on reasonable request

## Abbreviations

ANC: Antenatal care
BDT: Bangladeshi Taka
CAPI: Computer-assisted personal interviewing
CSBA: Community skilled birth attendant
DGNM: Directorate General of Nursing and Midwifery
FGD: Focus group discussion
FWV: Family welfare visitor
GBP: Great Britain Pounds
ICM: International Confederation of Midwives
MoHFW: Ministry of Health and Family Welfare
PNC: Postnatal care
SBA: Skilled birth attendants
TBA: Traditional birth attendant
TTBA: Trained traditional birth attendant
UNFPA: United Nations Population Fund
